# Impact of dining out frequency on the risk of colorectal cancer: insights from a large Chinese cohort

**DOI:** 10.3389/fonc.2025.1626303

**Published:** 2025-09-24

**Authors:** Pei Zhang, Wen-Jing Xing, Jing Zhang, Yin-Di Sun

**Affiliations:** ^1^ No.1 Ward Area of Oncology Department of East Campus, Zibo Central Hospital, Zibo, China; ^2^ Anorectal Ward Area of Traditional Chinese Medicine Department of East Campus, Zibo Central Hospital, Zibo, China

**Keywords:** dinning out, colon cancer, rectal cancer, colorectal cancer, obesity

## Abstract

**Introduction:**

Dining out has been shown to be associated with various negative health outcomes. However, the evidence concerning the relationship between dining out of home and the risk of colon and rectal cancers remains limited.

**Methods:**

We included a total of 42,286 participants aged between 20 and 60 years in this study, who underwent physical examinations at five large public hospitals located in Zibo, Shandong Province, China, from 2010 to 2022. Cox regression models were utilized to evaluate the association between the frequency of dining out and the risk of colon and rectal cancers, employing hazard ratios (HR) along with their corresponding 95% confidence intervals (CI). Restricted cubic spline (RCS) functions were applied to estimate the dose-response relationship. Subgroup analyses and sensitivity analyses were conducted to assess the robustness of the Cox regression models.

**Results:**

During a median follow-up of 10.3 years, this cohort study identified 272 new cases of colon cancer and 181 new cases of rectal cancer. After adjusting for confounding factors, frequent dining out was linked to an increased risk of both cancers, with HR of 2.231 (95% CI = 1.656-3.007) for colon cancer and 1.793 (95% CI=1.231-2.611) for rectal cancer compared to those who dined out rarely or never. The non-linear dose-response relationship between the frequency of dining out and the incidence of colon and rectal cancers demonstrated a significant pattern. Furthermore, obesity significantly mediated the associations between dining out frequency and the risks of developing both cancers.

**Discussion:**

Dining out frequently was significantly linked to an increased risk of colon and rectal cancer. Notably, obesity may partially mediate this relationship.

## Introduction

Cancer represented a significant societal, public health, and economic challenge in the 21st century. In 2022, over 1.9 million new colorectal cancer cases (including anal cancers) and about 904,000 deaths were estimated worldwide. This represents nearly one in ten of all global cancer cases and deaths (1). Colorectal cancer ranked among the top five cancers in terms of both incidence and mortality rates in China (2). Data from 1990 to 2021 indicated an upward trend in the incidence, mortality, prevalence, and disability-adjusted life years (DALYs) associated with colorectal cancer across both sexes and all age groups in China (3).

As modern life continues to accelerate, a notable rise in the frequency of dining out has emerged as a crucial element of evolving global eating habits. The National Health and Nutrition Examination Survey (NHANES) reported that from 2005 to 2014, 34% of individuals dined out. This figure rose to 64% among those aged 20 and over between 2017 and 2018 (4). In the United Kingdom, data for adults aged 19 and above showed that 27.1% dined out during 2008-2012; within this group, about 21.1% ordered takeaway at least once a week (5, 6). In Japan, the percentage of adults aged 20 years or older who dined out weekly increased from 32.3% in 2015 to 33.6% in 2019 (7). Similarly, among Chinese adults aged between 18–44 years and those aged between 45–59 years, weekly dining-out rates rose from 19.5% and 11.1%, respectively, in 2002 to 41.3% and 24.3%, respectively, in 2015 (8). Dining out has been found to have a significant negative effect on health outcomes, including overweight (9), inflammatory bowel disease (10), hyperuricemia (11), metabolic syndrome (12), tooth loss (13), and even mortality (14). Specifically, available evidence indicates that these adverse health outcomes are potentially associated with increased energy intake and an imbalance in macro- and micronutrient consumption (31–34). Furthermore, it was well established that the etiology of colorectal cancers is significantly influenced by lifestyle choices and dietary habits (15, 16). However, there were currently no studies that investigate the relationship between this essential life habit of dining out and the risk of developing colorectal cancers.

To address this evidence gap, we presented analyses examining the associations between dining out and the incidence of colon and rectal cancer in a large cohort of general Chinese adults from 2010 to 2022. Additionally, we aimed to investigate the dose-response relationship between the frequency of dining out and the risk of developing incident colon and rectal cancer. To further explore this relationship, we conducted mediation analyses to examine potential mediating factors.

## Methods

### Study design and participants

We collected data from physical examinations conducted in five designated public hospitals located in Zibo, Shandong Province, China, during the periods of September 2010 and September 2022. The study included a total of 42,286 participants aged between 20 and 60 years who had completed comprehensive questionnaires prior to their physical examinations. These questionnaires were designed to collect data on the lifestyles and habits of the individuals. The annual physical examinations were organized by the respective companies or units, with 95% participants engaging in at least ten waves during the 12-year follow-up period. Importantly, none of the participants had been diagnosed with any form of cancer before their initial participation in the survey. The data collection process followed three standardized criteria: uniform methods, a consistent questionnaire, and strict quality control. Data were entered twice for accuracy, and datasets from different hospitals were cleaned systematically to ensure uniformity. The integrated individual data were then matched with the diagnostic outcomes of colon and rectal cancer, sourced from the medical insurance system of Zibo, utilizing individual identification card numbers.

This research received ethical approval from the Ethics Committee of the Zibo Central Hospital. Informed consent was obtained from all study participants. Procedures followed the Declaration of Helsinki and relevant regulations.

### Definitions of dining out frequency

Data were collected from the unified basic and individual habits information questionnaires administered between 2010 and 2022. The data collection process involved group oral training, after which individuals completed the questionnaires prior to their physical examinations. The questionnaire pertinent to our study included inquiries regarding the frequency of meals consumed at various dining locations over the preceding week (7 days).

“Dining out” was defined as respondents having eaten outside their home at least once in the past 7 days or consuming non-homemade food as regular meals. The frequency of dining out was assessed by asking, “During the past 7 days, how many meals did you eat away from home?” Dining locations encompassed home, working place/school dining halls, Chinese restaurants/Western restaurants (including fast food restaurants), takeout (including orders and boxed lunches), bakeries/cake shops/coffee shops, and other venues. The weekly frequency of eating meals away from home was categorized as Never/almost never, (fewer than 1 meal per week), Sometimes (1 to 3 meals per week), or Frequent (4 or more meals per week).

### Follow-up and definitions of colon and rectal cancer

The diagnosis of cancer and the corresponding time were accurately recorded in the medical insurance system of Zibo. Participants were followed up through record linkage, utilizing their unique national ID numbers. According to the International Classification of Diseases, 10th Edition (ICD-10), the primary outcomes of this study were colon cancer (ICD-10: C18), rectal cancer (including anal cancer) (C19-C20), and their combined cancer, referred to as colorectal cancer. Participants contributed person-years from enrollment until the occurrence of the outcome, loss to follow-up, or the end of the study period in September 2022, whichever came first.

### Definitions of covariates

Demographic and socioeconomic characteristics assessed in this study included age group, sex, drinking status, smoking status, educational level, work intensity, daily sitting duration, and financial condition. Physical measurements comprised body mass index (BMI), waist circumference (WC), systolic blood pressure (SBP), and diastolic blood pressure (DBP). Height was measured to the nearest 0.1 cm using a vertical stadiometer. Weight was measured with the subject not wearing footwear and recorded to the nearest 0.1 kg. WC was recorded horizontally at the level of the subject’s umbilicus to the nearest 0.1 cm. Blood pressure readings were calculated as the average of three separate measurements. Dietary habits were evaluated based on the frequency of daily consumption of fresh fruits, vegetables, eggs, meat, and milk. The regularity of meal patterns was also investigated in our study. Furthermore, participants self-reported their history of digestive tract diseases and cancer.

In detail, the age group was dichotomized into young and middle-aged (under 50 years) and elderly (50 years and above), in accordance with classifications for late-onset colorectal cancer and early-onset colorectal cancer (17, 18). Sex was categorized as male or female. Smoking status and drinking status were assessed through similar questions that inquired whether participants currently smoke or consume alcohol. Educational level was classified as low (below a university degree) or high (university degree or above). Working intensity was self-assessed by participants as either light intensity or heavy intensity. Daily sitting time was categorized as moderate (less than 4 hours) or long (4 hours or more). Financial condition was evaluated using the question, “Are all financial sources sufficient for your family?” Responses were recorded as yes or no. According to criteria established by the Working Group on Obesity in China (WGOC), body mass index (BMI) was classified as normal (<28 kg/m²) or obesity (≥28 kg/m²) (19). Based on the latest standards for defining abdominal obesity in China, waist circumference thresholds for diagnosing abdominal obesity were set at ≥85 cm for men and ≥80 cm for women (20). Hypertension was defined by SBP of ≥130 mmHg or DBP of ≥80 mmHg (21). Food consumption across all wave surveys included five major food groups: fruits, vegetables, meat, eggs, and milk; responses were classified into two categories: frequent and seldom. Regular meal patterns were investigated using the question “Did you consistently exhibit any of the following eating behaviors: skipping breakfast/night eating/emotional eating?” Answers were recorded as yes or no. The history of digestive tract diseases and cancer was documented with responses categorized as yes or no.

### Statistical analysis

Since the proportion of missing data for all variables was below 10%, we employed multiple imputation methods to address these missing values (22, 23). The multiple imputation approach provided a robust framework for accurately representing the relationships among variables. In accordance with the principles of multiple imputation, we utilized a logistic regression model to assess the mechanism underlying the missing data, and then generated five imputed datasets. Finally, we pooled the results using the Markov chain Monte Carlo method based on chained equations (24, 25).

The descriptive statistics of baseline characteristics were presented according to the frequency of dining out. Continuous variables were reported as means with 95% confidence intervals (CIs). Categorical variables are displayed as frequencies and percentages. Cox proportional hazards regression models were used to calculate hazard ratios (HRs) and 95% CIs. The backward stepwise regression method identified significant covariates for inclusion in the final models. These adjusted models assessed the relationship between dining out frequency and the risk of colon and rectal cancer. Model I was unadjusted; Model II adjusted for age, sex, smoking status, drinking status, education level, household income, BMI, WC, blood pressure, and family history of digestive tract diseases and cancer; Model III further adjusted for dietary habits such as fresh fruit and vegetable intake, meat consumption, egg intake, and milk consumption.

Cox proportional hazards regression models incorporating adjusted restricted cubic splines (RCS) were employed to investigate the potential nonlinear association between dining out frequency and the risk of incident colon and rectal cancer (26, 27). In accordance with existing evidence-based recommendations, we designated the never dining out as the reference value for all analyses concerning nonlinear associations. The optimization of nonlinear curve fitting was achieved by including three knots in the models, thereby mitigating accuracy reduction associated with overfitting (28).

In order to further validate the robustness of the correlation between dining out frequency and the incidence of colon and rectal cancer, we conducted a subgroup analysis by categorizing several potential covariates. These analyses included subgroups categorized by age group, sex, educational level, drinking status, smoking status, financial condition, daily sitting time, general obesity, abdominal obesity, and hypertension. Additionally, sensitivity analyses were performed to ensure the reliability of the results. On one hand, the first part of our sensitivity analysis compared results before and after applying multiple imputation. On the other hand, in two subsequent parts of our sensitivity analyses, we excluded participants with a history of digestive tract diseases or a family history of cancer to evaluate the robustness of our findings.

Mediation analysis was utilized to evaluate both the direct and indirect associations between the frequency of dining out, covariates, and the incidence of colon and rectal cancers. Specifically, we first identified the direct association between the frequency of dining out as predictor variables (X) and the onset of colon/rectal/colorectal cancers as the outcome variable (Y). Subsequently, we investigated the indirect association involving frequency of dining out (X), one of the covariates serving as a mediator (M), and colon/rectal/colorectal cancers (Y). This methodology has been extensively employed in previous studies to quantify mediation effects (29, 30). Given that both general and abdominal obesity have been demonstrated to be associated not only with dining out but also with the incidence of colorectal cancer in previous studies, this study aimed to investigate how obesity mediates the relationship between dining out and the occurrence of colorectal cancer (31–36).

All analyses were conducted utilizing R and SPSS software, with statistical significance assessed at a two-tailed p-value of less than 0.05.

## Results

### Missing data processing

As presented in **Supplementary Table 1**, the results of the logistic regression analysis between independent and dependent variables indicated that all p-values were non-significant. This finding suggests that the missing data in both the independent and dependent variables of this study are independent. Consequently, we assumed that the missing data were missing at random (MAR).

As illustrated in **Supplementary Figure 1**, after imputing the missing data, it appears that the distributions of the imputed and observed values are quite similar. Specifically, the observed data is represented in gray, while the imputed data is shown in red. The plot provides a clear comparison of values both before and after imputation.

### Baseline characteristics of study participants

A total of 42,286 participants aged between 20 and 60 years, all without any history of cancer, underwent physical examinations at five public hospitals in Zibo, Shandong Province, China from 2010 to 2022. This cohort study identified 272 new cases of colon cancer and 181 new cases of rectal cancer during a median follow-up period of 10.3 years. The study included 20,183 participants who rarely dined out, 14,867 participants who occasionally dined out, and 7,236 participants who frequently dined out.

The average ages of the participants who never dined out (40.0 years; 95%CI= 39.8-40.2), those who sometimes dined out (40.0 years; 95%CI=39.8-40.2), and those who often dined out (39.8 years; 95%CI=39.5-40.1) were similar across groups. Participants who frequently dine out demonstrated a higher prevalence of males, as well as individuals who consume alcohol and smoke. Additionally, this group exhibited greater intake of meat and milk compared to other groups. Among the total participants, it was found that only 91.2% could maintain regular meal patterns; however, this figure dropped to just 81% among those who frequently dined out (**Table 1**).

**Table 1:** Characteristics of participants based on the frequency of dinning out of home per week.

Characteristics	Total (N=42 286)	Never/almost never (N=20 183)*	Sometimes (N=14 867)*	Frequent (N=7 236)*	P-value
Age (years)	40.0 (39.9-40.1)	40.0 (39.8-40.2)	40.0 (39.8-40.2)	39.8 (39.5-40.1)	0.216
Male (%)	21693 (51.3%)	10138 (50.2%)	7525 (50.6%)	4030 (55.7%)	<0.001
Drinking status	6179 (14.6%)	2886 (14.3%)	2049 (13.8%)	1244 (17.2%)	<0.001
Smoking status	5360 (12.7%)	2451 (12.1%)	1831 (12.3%)	1078 (14.9%)	<0.001
High educational level	13987 (33.1%)	6659 (33.0%)	4960 (33.4%)	2368 (32.7%)	0.602
High working intensity	1518 (3.6%)	686 (3.4%)	565 (3.8%)	267 (3.7%)	0.120
Long time of sitting time	19006 (44.9%)	9096 (45.1%)	6643 (44.7%)	3267 (45.1%)	0.720
Good financial condition	5889 (13.9%)	2567 (12.7%)	1918 (12.9%)	1404 (19.4%)	<0.001
BMI	23.9 (23.9-24.0)	23.4 (23.4-23.5)	23.5 (23.5-23.6)	24.8 (24.8-24.9)	0.021
WC	81.0 (80.9-81.1)	81.0 (80.9-81.1)	80.9 (80.8-81.1)	81.1 (80.9-81.3)	0.138
DBP	79.9 (79.8-79.9)	79.9 (79.8-80.0)	79.9 (79.8-80.0)	79.8 (79.7-79.9)	0.212
SBP	132.5 (132.4-132.6)	132.5 (132.4-132.7)	132.5 (132.3-132.6)	132.4 (132.2-132.6)	0.347
Fruits	14128 (33.4%)	6760 (33.5%)	4924 (33.1%)	2444 (33.8%)	0.589
Vegetables	41575 (98.3%)	19830 (98.3%)	14617 (98.3%)	7128 (98.5%)	0.347
Meat	21170 (50.1%)	9807 (46.3%)	7376 (49.6%)	3987 (55.1%)	<0.001
Egg	31625 (74.8%)	15036 (74.5%)	11227 (75.5%)	5362 (74.1%)	0.032
Milk	8533 (20.2%)	3540 (17.5%)	3177 (21.4%)	1816 (25.1%)	<0.001
Regular eating meals	38586 (91.2%)	19315 (95.7%)	13410 (90.2%)	5861 (81.0%)	<0.001
History of digestive tract diseases	1714 (4.1%)	826 (4.1%)	618 (4.2%)	270 (3.7%)	0.298
Family history of cancer	5383 (12.7%)	2515 (12.5%)	1920 (12.9%)	948 (13.1%)	0.263

N, number of participants; BMI, body mass index; WC, waist circumstance; SBP, systolic blood pressure; DBP, diastolic blood pressure.

*The weekly frequency of dinning out of home is categorized as: Never/almost never (fewer than one meal per week), Sometimes (one to three meals per week), and Frequent (four or more meals per week).

Continuous variables were displayed as means with 95% confidence intervals. Categorical variables were displayed as frequencies and percentages.

### Association between the frequency of dining out and colorectal cancer

The relationship between the frequency of dining out and the risk of colon, rectal, and colorectal cancers was examined using Cox proportional hazards regression models adjusted for covariates. (**Table 2**) Participants who dined out occasionally exhibited a 1.505-fold increased risk of developing incident colon cancer (HR=1.505, 95% CI=1.144-1.979) compared to those who never or rarely dined out per week. In contrast, participants who frequently dined out demonstrated a 2.231-fold increased risk (HR=2.231, 95% CI=1.656-3.007) for the same outcome. Similarly, participants who dined out frequently exhibited a 1.793-fold (HR=1.793, 95% CI=1.231-2.611) increased risk of incident rectal cancer relative to those who never or rarely dined out per week. When combining the outcomes of colon and rectal cancers into colorectal cancers, we observed that participants who dined out occasionally faced a 1.564-fold higher risk of incident colorectal cancers (HR=1.564, 95% CI=1.258-1.945), while those who often dined out had a significantly elevated risk with an HR of 2.265 (95% CI=1.784-2.875), compared to individuals who never or rarely engaged in dining out on a weekly basis.

**Table 2:** Adjusted hazard ratios for the risk of colon, rectal, and colorectal cancer based on dining out frequency.

Outcomes	Model I HR (95% CI)	Model II HR (95% CI)	Model III HR (95% CI)
Colon cancer			
Never/almost never	Reference	Reference	Reference
Sometimes	1.504 (1.144-1.977)	1.504 (1.144-1.978)	1.505 (1.144-1.979)
Frequent	2.230 (1.655-3.004)	2.225 (1.651-2.998)	2.231 (1.656-3.007)
P-value	0.003	<0.001	<0.001
Rectal cancer			
Never/almost never	Reference	Reference	Reference
Sometimes	1.298 (0.927-1.816)	1.295 (0.925-1.812)	1.290 (0.922-1.806)
Frequent	1.801 (1.237-2.622)	1.791 (1.230-2.608)	1.793 (1.231-2.611)
P-value	0.009	0.010	0.012
Colorectal cancers ^a^			
Never/almost never	Reference	Reference	Reference
Sometimes	1.570 (1.262-1.952)	1.567 (1.260-1.949)	1.564 (1.258-1.945)
Frequent	2.272 (1.790-2.883)	2.262 (1.782-2.872)	2.265 (1.784-2.875)
P-value	<0.001	<0.001	<0.001

HR, hazard ratio; CI, confidential interval.

Model I: Crude HR (95% CI).

Model II: adjusted for stratification by age and sex, as well as for drinking status, smoking status, education level, working intensity, daily sitting time, financial condition, BMI, WC, blood pressure, condition of regular eating meals, history of digestive tract diseases and family history of cancer.

Model III: additionally adjusted for dietary habits (consumption frequency of fresh fruits and vegetables, meat, egg, and milk).

a This endpoint is the first incident colorectal cancers (which could be either colon or rectal cancer).

**Figure 1:**
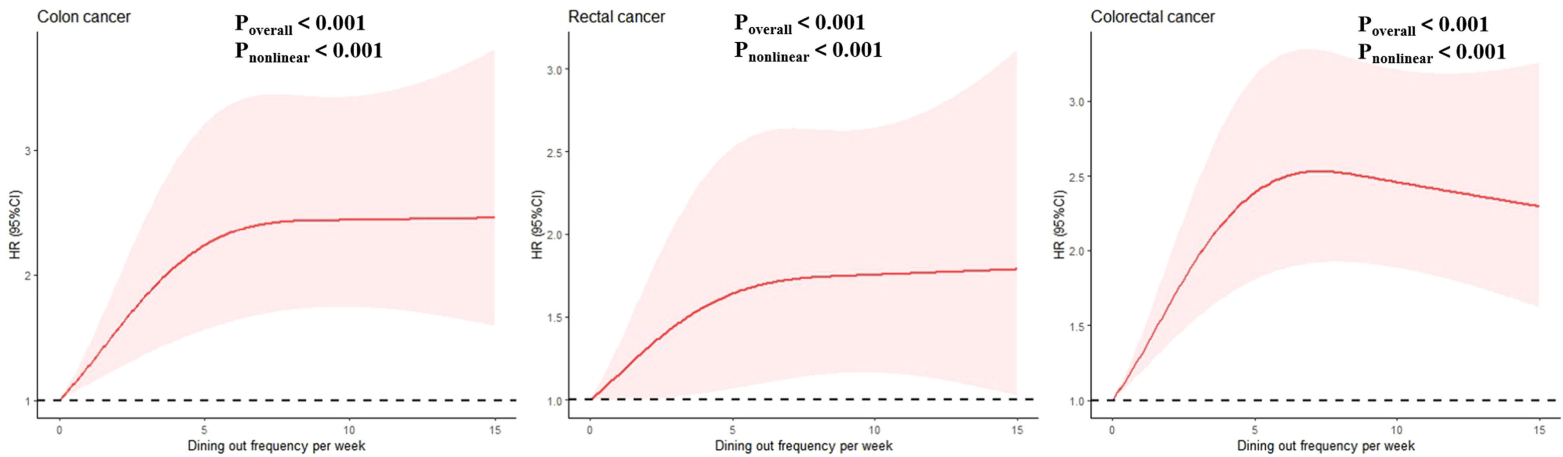
Nonlinear association between frequency of dinning out with risk of colon, rectal, coloretal cancer among total participants. Associations were evaluated utilizing multivariable Cox regression models incorporating restricted cubic splines.

A non-linear relationship between the frequency of dining out and the risk of colon, rectal, and colorectal cancers is depicted in **Figure 1**. A statistically significant association exists between the frequency of dining out and the risk of these cancers. Further studies conducted in both sexes have observed similar results, as illustrated in **Supplementary Figures 2**, **3**.

### Subgroup and sensitivity analyses

**Table 3** presents the results of serial subgroup analyses stratified by age, sex, educational level, drinking status, smoking status, financial condition, daily sitting time, BMI, WC, and blood pressure. The subgroup analyses did not indicate any significant alterations compared to the overall analysis. These analyses confirmed that individuals who frequently dine out have a higher likelihood of developing incident colon, rectal, and colorectal cancers compared to those who dine out never or almost never per week.

**Table 3:** Subgroup analysis of adjusted hazard ratios for the risk of colon, rectal, and colorectal cancer based on frequency of dining out.

Characteristics	Subgroups	Colon cancer (HR (95% CI))			Rectal cancer (HR (95% CI))			^a^ Colorectal cancers (HR (95% CI))		
		Never	Sometimes	Frequent	Never	Sometimes	Frequent	Never	Sometimes	Frequent
Age group	< 50 years old	1.000	1.622(1.078-2.440)	1.657(1.072-2.562)	1.000	1.145(0.709-1.851)	1.723(0.989-3.003)	1.000	1.401(1.027-1.910)	2.058(1.440-2.940)
	≥ 50 years old	1.000	1.410(0.975-2.040)	2.973(1.957-4.517)	1.000	1.459(0.910-2.340)	1.874(1.125-3.123)	1.000	1.748(1.286-2.377)	2.468(1.789-3.404)
Sex	female	1.000	1.728(1.195-2.499)	2.237(1.480-3.381)	1.000	1.263(0.815-1.958)	1.255(0.729-2.163)	1.000	1.665(1.248-2.222)	1.970(1.413-2.746)
	male	1.000	1.260(0.835-1.901)	2.224(1.446-3.422)	1.000	1.349(0.799-2.279)	2.635(1.544-4.496)	1.000	1.451(1.040-2.023)	2.677(1.898-3.778)
Educational level	low	1.000	1.934(1.384-2.703)	2.363(1.616-3.457)	1.000	1.331(0.868-2.042)	1.793(1.106-2.908)	1.000	1.331(0.868-2.042)	1.793(1.106-2.908)
	high	1.000	0.861(0.521-1.423)	2.030(1.255-3.284)	1.000	1.245(0.723-2.145)	1.796(0.989-3.261)	1.000	1.203(0.822-1.762)	2.286(1.551-3.369)
Drinking status	yes	1.000	1.519(1.129-2.043)	2.273(1.647-3.135)	1.000	1.828(0.678-4.932)	3.115(1.123-8.637)	1.000	2.115(1.121-3.989)	3.216(1.636-6.319)
	no	1.000	1.428(0.697-2.929)	1.930(0.866-4.303)	1.000	1.231(0.860-1.761)	1.231(0.860-1.761)	1.000	1.499(1.188-1.891)	2.137(1.655-2.759)
Smoking status	yes	1.000	1.578(1.178-2.115)	2.365(1.720-3.251)	1.000	1.440(1.004-2.064)	1.849(1.226-2.788)	1.000	1.676(1.327-2.116)	2.371(1.834-3.065)
	no	1.000	1.065(0.482-2.353)	1.447(0.605-3.459)	1.000	0.547(0.191-1.570)	1.496(0.581-3.849)	1.000	0.926(0.488-1.757)	1.649(0.857-3.176)
Financial condition	good	1.000	1.496(1.116-2.005)	2.072(1.498-2.866)	1.000	1.312(0.913-1.885)	1.812(1.209-2.716)	1.000	1.550(1.228-1.956)	2.144(1.657-2.774)
	so so/bad	1.000	1.550(0.717-3.351)	3.400(1.573-7.353)	1.000	1.214(0.493-2.990)	1.736(0.631-4.777)	1.000	1.726(0.931-3.199)	3.281(1.735-6.205)
Daily sitting time	moderate	1.000	1.306(0.888-1.922)	1.794(1.165-2.765)	1.000	1.362(0.849-2.183)	1.526(0.871-2.676)	1.000	1.675(1.218-2.302)	2.131(1.488-3.052)
	long	1.000	1.942(1.270-2.971)	2.753(1.734-4.370)	1.000	1.116(0.656-1.897)	1.992(1.149-3.454)	1.000	1.434(1.040-1.978)	2.207(1.562-3.119)
General obesity	yes	1.000	1.526(0.856-2.723)	2.349(1.260-4.383)	1.000	1.901(0.873-4.140)	4.080(1.893-8.794)	1.000	1.806(1.122-2.908)	3.207(1.963-5.241)
	no	1.000	1.500(1.099-2.046)	2.202(1.568-3.092)	1.000	1.184(0.814-1.721)	1.374(0.880-2.145)	1.000	1.510(1.182-1.929)	2.041(1.552-2.685)
Abdominal obesity	yes	1.000	1.650(1.026-2.651)	2.208(1.311-3.719)	1.000	0.799(0.43-1.482)	1.168(0.592-2.307)	1.000	1.485(1.009-2.187)	2.047(1.340-3.127)
	no	1.000	1.435(1.027-2.007)	2.236(1.555-3.216)	1.000	1.618(1.076-2.435)	2.225(1.410-3.510)	1.000	1.612(1.239-2.099)	2.390(1.791-3.190)
Hypertension	yes	1.000	1.470(0.866-2.494)	2.069(1.144-3.740)	1.000	1.195(0.645-2.215)	1.949(1.003-3.789)	1.000	1.500(0.994-2.262)	2.242(1.428-3.521)
	no	1.000	1.480(1.076-2.036)	2.233(1.582-3.152)	1.000	1.343(0.898-2.008)	1.747(1.108-2.755)	1.000	1.590(1.229-2.057)	2.275(1.717-3.014)

HR, hazard ratio; CI, confidential interval.

a This endpoint is the first incident colorectal cancers (which could be either colon or rectal cancer).

To validate the robustness of the primary findings, three sensitivity analyses were conducted, as detailed in **Table 4**. First, the results remained consistent across both the complete data set without missing values (3,925 participants) and the multiple imputed data sets. Second, excluding participants with a history of digestive tract diseases at baseline yielded similar outcomes. Third, after removing participants with a family history of cancer at baseline, dining out continued to show a significant association with the risk of incident colorectal cancers.

**Table 4:** Sensitive analyses of adjusted hazard ratios for the risk of colon, rectal, and colorectal cancer based on dining out frequency.

Outcomes	Participants with complete data [HR (95% CI)]	Participants without digestive tract disease history [HR (95% CI)]	Participants without family cancer history [HR (95% CI)]
	N=38,361	N=40,572	N=36,903
Colon cancer			
Never/almost never	Reference	Reference	Reference
Sometimes	1.499(1.132-1.985)	1.538(1.162-2.035)	1.431(1.067-1.918)
Frequent	2.225(1.640-3.017)	2.282(1.684-3.093)	2.258(1.649-3.094)
P-value	<0.001	<0.001	<0.001
Rectal cancer			
Never/almost never	Reference	Reference	Reference
Sometimes	1.286(0.912-1.814)	1.294(.920-1.820)	1.404(0.973-2.028)
Frequent	1.785(1.218-2.617)	1.796(1.228-2.626)	1.850(1.221-2.802)
P-value	<0.001	0.010	0.013
Colorectal cancers *			
Never/almost never	Reference	Reference	Reference
Sometimes	1.590(1.270-1.991)	1.580(1.265-1.972)	1.602(1.265-2.029)
Frequent	2.301(1.802-2.939)	2.286(1.794-2.912)	2.364(1.828-3.057)
P-value	<0.001	<0.001	<0.001

HR, hazard ratio; CI, confidential interval; N, number of participants.

*This endpoint is the first incident colorectal cancers (which could be either colon or rectal cancer).

### Mediation analysis

**Figure 2:**
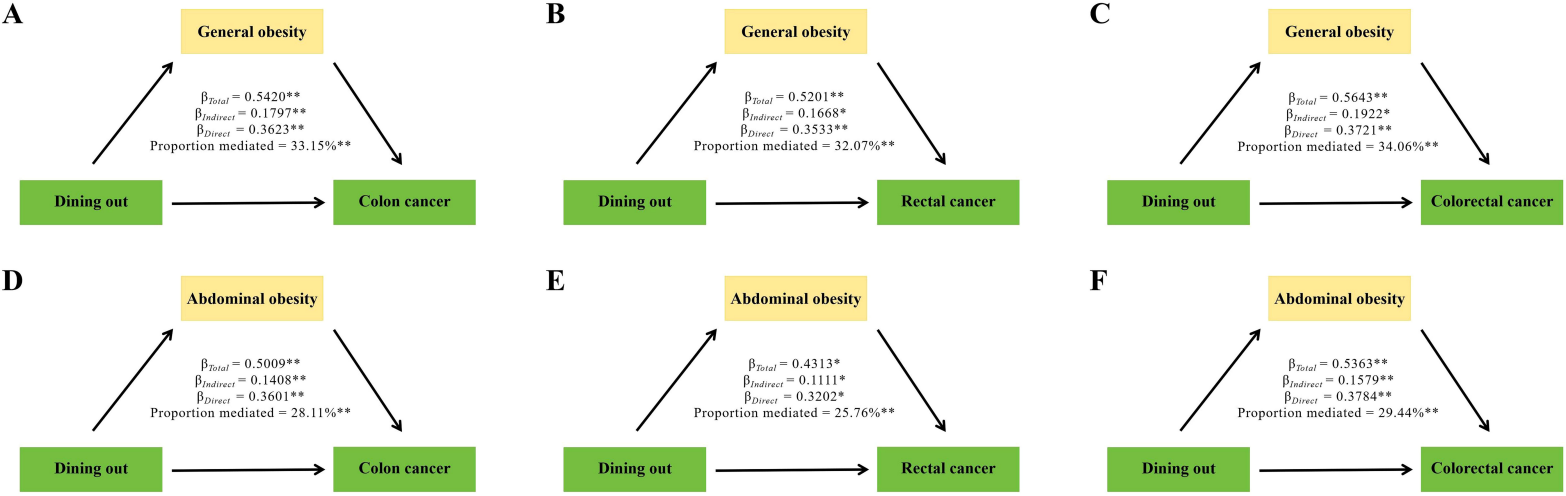
Mediation analysis was conducted to examine the effects of general **(A–C)** and abdominal obesity **(D–F)** on the relationship between dining out and the risk of developing colon, rectal, and colorectal cancer. *P<0.05, **P<0.01.

**Figure 2** summarized the parallel mediation analyses conducted to the mediating effects of general and abdominal obesity on the relationship between dining out frequency and colon, rectal, and colorectal cancer. After conducting fully adjusted analyses, the significant mediation proportions of general obesity were found to be 33.68%, 32.07%, and 34.66% in relation to the associations between dining out and colon, rectal, and colorectal cancer, respectively. Similarly, the significant mediation proportions for abdominal obesity were observed at 28.12%, 25.75%, and 25.44%. These results suggested that obesity may play a role in the development of colorectal cancer by partially mediating the effects associated with dining out.

## Discussion

Our findings indicated that over half of the Chinese adult population reported dining out at least once per week. Furthermore, a significant association was observed between frequent dining out and an elevated risk of both colon and rectal cancers when compared to individuals who dined out rarely or never. The relationship between the frequency of dining out and the incidence risk of colon and rectal cancers exhibited a pronounced dose-response association pattern. Additionally, obesity served as a significant mediator in the associations between dining out and the risks associated with developing both types of cancer.

Given studies suggested that the etiology of colorectal cancers encompasses both genetic and environmental factors. Among colorectal cancer cases, only approximately 20% can be attributed to heritable gene variations (37), indicating that the majority of sporadic colorectal cancer cases were associated with environmental causes (38). Among the environmental factors influencing the risk of developing colorectal cancers, dietary parameters were believed to play a significant role (39). Participants who had frequent dinning out per week in our results had a higher proportion of smoking, drinking alcohol, meat and milk intake. Similar findings have been reported in previous studies, which have identified several key dietary and lifestyle factors associated with colorectal cancer. Smoking and alcohol consumption is a well-recognized risk factor for colorectal cancer (40). Li et al. reported that smoking is a strong risk factor for colorectal cancer regardless of current smokers or former smokers (41). Chen et al. found that lifetime average alcohol consumption even 25 g/d was strongly associated with colorectal cancer risk (42). As a big alcohol consumer in China, especially when eating out, people are used to socialize with each other through drinking. Therefore, eating out indirectly promoted the alcohol intake of Chinese people (43). A similar trend has been observed in Mediterranean and Western European contexts, where dining outside the home is associated with higher levels of alcohol intake, especially on weekends (44). Dining out has always been associated with higher intake of red and processed meats, which are clearly recognized as risk factors for the incidence of colorectal cancers (45). Most studies emphasize that meat processing significantly increases colorectal cancer risk. This is due to the conversion of nitrates and nitrites in processed meat into N-nitroso-compounds, which can form covalent adducts with DNA bases, ultimately leading to colorectal cancer development (46, 47). Additionally, frequent dining out for breakfast was found to be associated with an increased intake of milk within the context of the Chinese dietary pattern for breakfast. Previous meta-analyses have supported an inverse relationship between non-fermented milk consumption and the risk of colon cancer, attributing this association to higher energy and fat intake linked to milk consumption (48).

Dining out as a novel environmental factor linked to colorectal cancers were found in our study for the first time. The underlying mechanism may be associated with the consistent links between dining out and weight gain (9). Prior research has demonstrated that eating out or consuming meals away from home is significantly correlated with increased energy intake and nutrient deficiencies, which can subsequently contribute to weight gain. Specifically, a systematic review indicated that eating outside the home was associated with a greater total energy intake and a higher proportion of energy derived from fat in the daily diet (31). Additionally, the energy contribution from consuming food outside the home constituted more than half of the daily energy intake in various instances. Furthermore, dining out was associated with lower intakes of micronutrients, particularly vitamin C, calcium (Ca), and iron (Fe) (32). Notably, subsequent large-scale systematic reviews have indicated that a higher consumption of foods purchased outside the home was associated with increased intakes of energy and nutrients. Moreover, individuals who frequently consume dining-out food tend to have higher fat intake across various dietary patterns (33). Numerous epidemiological studies suggested that approximately 11% of colorectal cancers can be attributed to overweight and obesity; specifically, each 1 kg/m²increase in BMI confers an additional risk (HR=1.03, 95% CI=1.01-1.05). However, visceral fat or abdominal obesity seems to be of similar concern like subcutaneous fat obesity (34). A working group from the International Agency for Research on Cancer reviewed over 1,000 epidemiological studies and reported that the risk of colorectal cancer increased by a factor of 1.2-1.5 among patients with a BMI exceeding 25 kg/m², and by a factor of 1.5-1.8 in individuals with a BMI ≥30 kg/m². A similar association was observed between WC, particularly when comparing the highest versus lowest categories (35). Furthermore, a meta-analysis including 56 studies revealed a dose-response relationship between five BMI categories (<23.0, 23.0-24.9, 25.0-27.4, 27.5-29.9, and >30.0 kg/m²) and an increased risk of colorectal cancer: with hazard ratios of 1.0 (reference), 1.14 (95%CI=1.06-1.23), 1.19 (95%CI=1.13-1.25), 1.24 (95%CI=1.15-1.35), and 1.41 (95%CI=1.30-1.53). These findings indicated that the risk of colorectal cancer escalates with overweight or obesity status, irrespective of the specific parameter assessed—be it BMI, waist circumference, weight gain, or other metrics (36). These findings may elucidate the mediating role of obesity including general obesity and abdominal obesity, in the relationship between frequent dining out and the risk of colorectal cancer to some extent.

Although menu labeling and nutrition profiling systems have been implemented, they have shown significant value in monitoring the nutritional profiles of foods that are offered and purchased. Their adoption could contribute to a reduction in unhealthy outcomes associated with dining out (49). However, Thaisa et al. demonstrated that menu labeling for away-from-home dining did not result in significant changes in either the quantity or quality of carbohydrate, total fat, saturated fat, or sodium intake within their study (50). Therefore, national governments should incorporate mechanisms to regularly monitor what is offered and consumed when dining out from new perspectives.

The strengths of our study include its prospective design, a large number of cases, the assessment of multiple aspects related to the frequency of dining out, and adjustments for a wide range of confounders. However, several limitations should be acknowledged. First, the frequency of dining out was self-reported, which may introduce recall bias and potentially result in either underreporting or overreporting. Secondly, although we incorporated numerous covariates associated with colorectal cancer in our analyses, there may still be uncontrolled and unmeasured confounders affecting the causal chain. Additionally, data on the quantity of dining out frequency were not available for more accurate quantification of the observed relationships or investigation into potential threshold effects. Then, The baseline frequency of dining out was utilized to evaluate the association between dining out and the risk of colorectal cancer. However, upon conducting additional studies, we discovered that the ICC values for dining out frequency from 2010 through subsequent years (2011 to 2022) ranged from 0.71 to 0.83. These high ICC values indicate that individuals’ dining frequencies at baseline (2010) remained quite consistent in the following years up until 2022. Finally, all participants were drawn from a population undergoing physical examinations of employed individuals. This limitation restricted our data collection to individuals under 60 years old (the legal retirement age in China) who are currently employed. Therefore, caution is warranted when comparing our results with those from other studies.

In conclusion, the findings of our study demonstrate that frequent dining out is significantly associated with an elevated risk of colorectal cancer. Additionally, obesity may partially mediate this relationship. Therefore, it is crucial to implement policies and initiatives aimed at monitoring and addressing these unhealthy lifestyle habits to safeguard public health.

## Data Availability

The data analyzed in this study is subject to the following licenses/restrictions: The raw data of this article will be made available by the corresponding author. Requests to access these datasets should be directed to Yin-Di Sun, walle_eva5886@163.com.

## Supplementary material

Supplementary Figure 1 Results of distributions of the imputed and observed values. The observed data is represented in gray, whereas the imputed data is shown in red.

Supplementary Figure 2 Nonlinear association between frequency of dinning out with risk of colon, rectal, coloretal cancer among male participants. Associations were evaluated utilizing multivariable Cox regression models incorporating restricted cubic splines.

Supplementary Figure 3 Nonlinear association between frequency of dinning out with risk of colon, rectal, coloretal cancer among female participants. Associations were evaluated utilizing multivariable Cox regression models incorporating restricted cubic splines.
