# Supplementary figures and images for: Impact of dining out frequency on the risk of colorectal cancer: insights from a large Chinese cohort
Source: Front Oncol. 2025 Sep 24;15:1626303. doi: 10.3389/fonc.2025.1626303 (PMC12504089; doi:10.3389/fonc.2025.1626303)

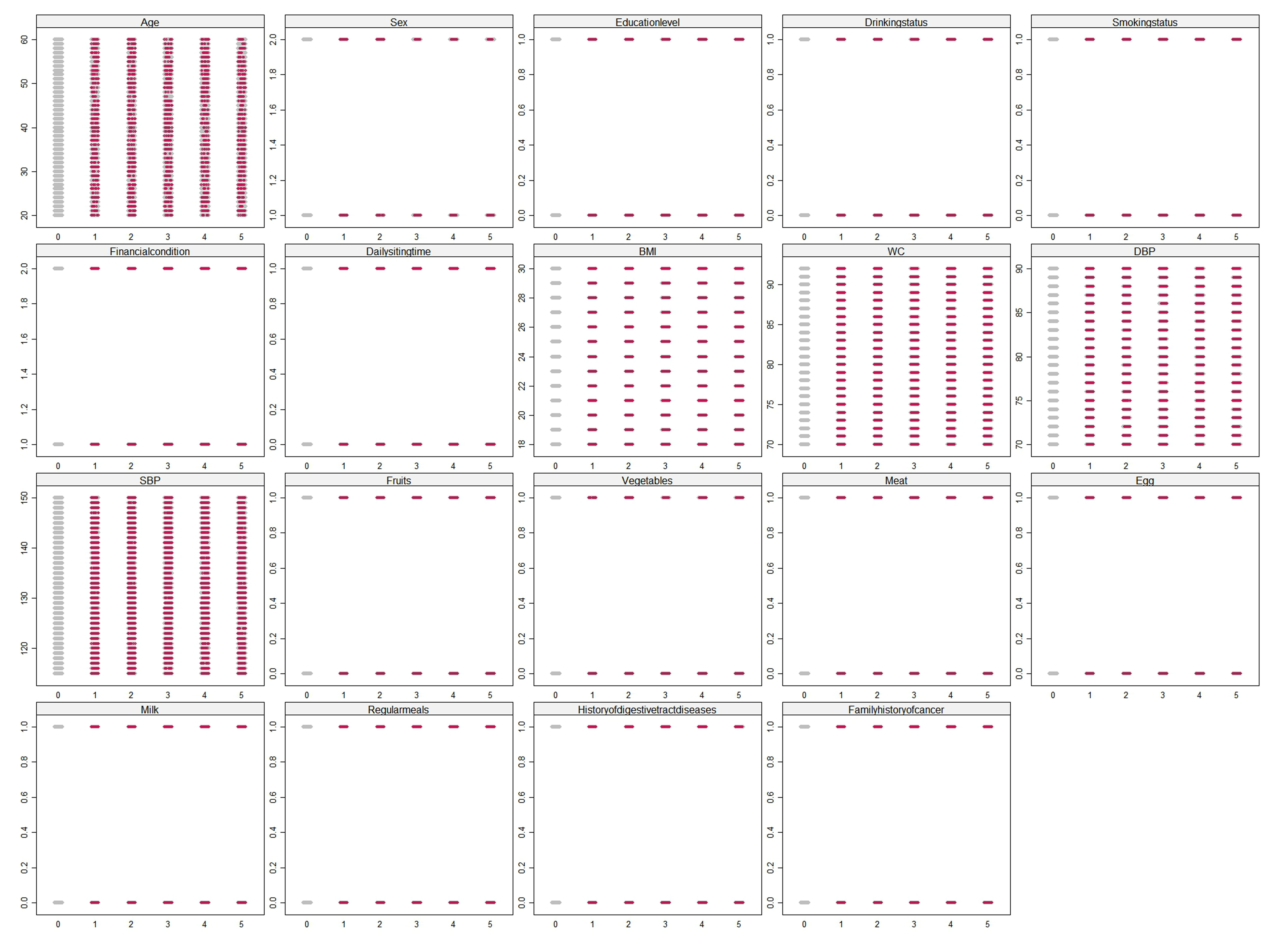

Supplement: Supplementary Figure 1 — Results of distributions of the imputed and observed values. The observed data is represented in gray, whereas the imputed data is shown in red. [file Image1.jpeg]

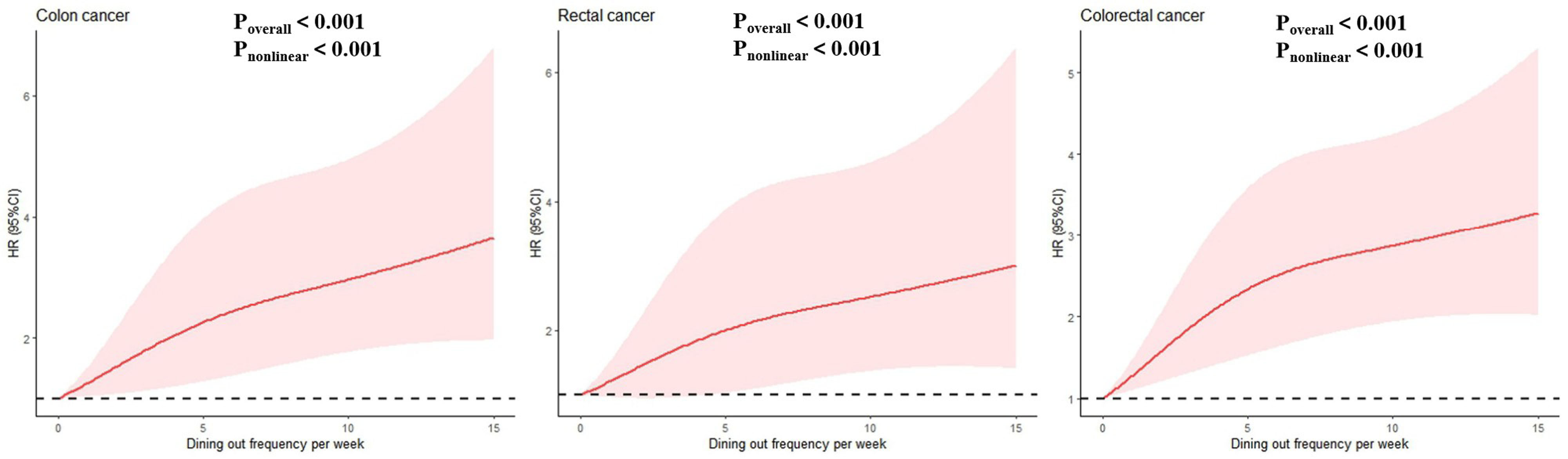

Supplement: Supplementary Figure 2 — Nonlinear association between frequency of dinning out with risk of colon, rectal, coloretal cancer among male participants. Associations were evaluated utilizing multivariable Cox regression models incorporating restricted cubic splines. [file Image2.jpeg]

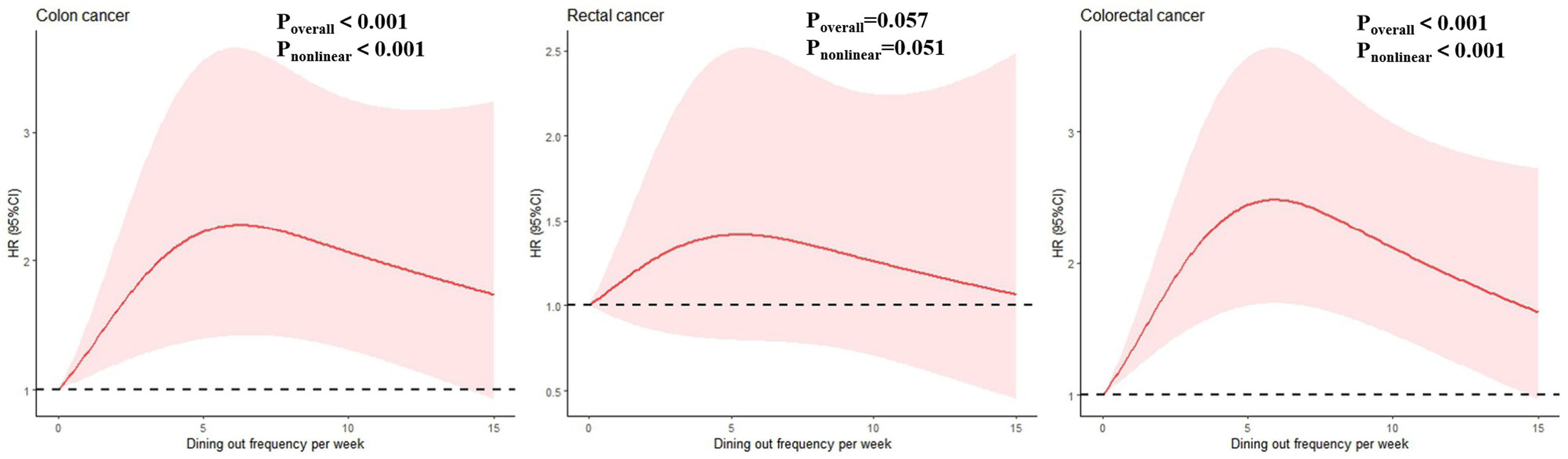

Supplement: Supplementary Figure 3 — Nonlinear association between frequency of dinning out with risk of colon, rectal, coloretal cancer among female participants. Associations were evaluated utilizing multivariable Cox regression models incorporating restricted cubic splines. [file Image3.jpeg]
