# Supplementary material for: Impact of dining out frequency on the risk of colorectal cancer: insights from a large Chinese cohort
Source: Front Oncol. 2025 Sep 24;15:1626303. doi: 10.3389/fonc.2025.1626303 (PMC12504089; doi:10.3389/fonc.2025.1626303)
Supplement: Supplementary file 4 [file Table1.docx]

**Supplementary table 1.** The results of logistic regression model (p-values) in estimating the mechanism of missing data.

| Outcomes/variables | Age | Educational  level | Working  intensity | Drink | Smoke | Financial condition | Daily sitting time | BMI | WC | SBP | DBP | Fruits | Vegetables | Meat | Eggs | Milk | Regular meals | History of digestive diseases | Family history of cancer | Frequency of dinning out |
| --- | --- | --- | --- | --- | --- | --- | --- | --- | --- | --- | --- | --- | --- | --- | --- | --- | --- | --- | --- | --- |
| Colon cancer | 0.579 | 0.131 | 0.438 | 0.615 | 0.383 | 0.787 | 0.63 | 0.741 | 0.205 | 0.684 | 0.782 | 0.830 | 0.484 | 0.162 | 0.505 | 0.710 | 0.700 | 0.121 | 0.311 | 0.567 |
| Rectal cancer | 0.065 | 0.837 | 0.759 | 0.471 | 0.772 | 0.105 | 0.239 | 0.585 | 0.186 | 0.546 | 0.701 | 0.714 | 0.641 | 0.292 | 0.279 | 0.786 | 0.235 | 0.553 | 0.126 | 0.142 |
| Colorectal cancer | 0.282 | 0.305 | 0.713 | 0.201 | 0.295 | 0.448 | 0.283 | 0.269 | 0.423 | 0.816 | 0.139 | 0.489 | 0.338 | 0.687 | 0.484 | 0.358 | 0.199 | 0.660 | 0.705 | 0.308 |
